# Predation on an Upper Trophic Marine Predator, the Steller Sea Lion: Evaluating High Juvenile Mortality in a Density Dependent Conceptual Framework

**DOI:** 10.1371/journal.pone.0030173

**Published:** 2012-01-17

**Authors:** Markus Horning, Jo-Ann E. Mellish

**Affiliations:** 1 Department of Fisheries and Wildlife, Marine Mammal Institute, Oregon State University, Newport, Oregon, United States of America; 2 Coastal Oregon Marine Experiment Station, Newport, Oregon, United States of America; 3 Alaska SeaLife Center, Seward, Alaska, United States of America; 4 School of Fisheries and Ocean Sciences, University of Alaska Fairbanks, Alaska, United States of America; Institut Pluridisciplinaire Hubert Curien, France

## Abstract

The endangered western stock of the Steller sea lion (*Eumetopias jubatus*) – the largest of the eared seals – has declined by 80% from population levels encountered four decades ago. Current overall trends from the Gulf of Alaska to the Aleutian Islands appear neutral with strong regional heterogeneities. A published inferential model has been used to hypothesize a continuous decline in natality and depressed juvenile survival during the height of the decline in the mid-late 1980's, followed by the recent recovery of juvenile survival to pre-decline rates. However, these hypotheses have not been tested by direct means, and causes underlying past and present population trajectories remain unresolved and controversial. We determined post-weaning juvenile survival and causes of mortality using data received post-mortem via satellite from telemetry transmitters implanted into 36 juvenile Steller sea lions from 2005 through 2011. Data show high post-weaning mortality by predation in the eastern Gulf of Alaska region. To evaluate the impact of such high levels of predation, we developed a conceptual framework to integrate density dependent with density independent effects on vital rates and population trajectories. Our data and model do not support the hypothesized recent recovery of juvenile survival rates and reduced natality. Instead, our data demonstrate continued low juvenile survival in the Prince William Sound and Kenai Fjords region of the Gulf of Alaska. Our results on contemporary predation rates combined with the density dependent conceptual framework suggest predation on juvenile sea lions as the largest impediment to recovery of the species in the eastern Gulf of Alaska region. The framework also highlights the necessity for demographic models based on age-structured census data to incorporate the differential impact of predation on multiple vital rates.

## Introduction

The endangered, western population segment of the Steller sea lion (*Eumetopias jubatus*) has declined to about 20 percent of peak levels recorded four decades ago, with locally divergent but overall stable trends in the Gulf of Alaska (GOA) and Bering Sea - Aleutian Islands (BSAI) [1]. The formerly less abundant, threatened eastern population (east of 144° West longitude) has increased about 3% *p.a.* during this period from South-east Alaska through California [1], [2]. Other upper trophic level mesopredators in the GOA and BSAI, including northern fur seals (*Callorhinus ursinus*), harbor seals (*Phoca vitulina*) and sea otters (*Enhydra lutris*) have also exhibited precipitous declines through portions of their range [3]–[7].

Several hypotheses describing forcing on these mesopredators in the GOA – BSAI region have been advanced, including the resource-driven junk food [8] and ocean climate [9] hypotheses, and the consumer-driven sequential megafaunal collapse hypothesis. The latter suggests a cascading prey shift in transient killer whales (*Orcinus orca)* triggered by the collapse of their former prey, the great whales, through commercial whaling [3], [10].

Resource driven hypotheses primarily postulate changes in abundance, distribution and accessibility, composition or nutritional quality of prey [8], [11]. These changes may be natural (i.e. driven by episodic changes in ocean climate) or anthropogenic (i.e. through large scale industrial fishing). Such bottom-up effects are thought to reduce fitness primarily through negative impacts on overall energy budgets of individual animals. When energetic demands associated with homeostasis or growth, foraging and reproduction are not balanced by energy gained through prey consumption, the ability to grow, survive or reproduce is compromised. Consumer driven hypotheses postulate direct causes of individual animal mortality independent of energetic balance [8], [11]. Predation, incidental mortality in fishing gear, ship strikes, subsistence harvest and illegal shooting comprise such top-down forces [8], [11], [12]. Pollutants and diseases may fall into either category. Lethal pathogens and pollutants can directly affect survival, while non-lethal agents can indirectly affect growth and reproduction through altered energy balance. Pollutants can also directly affect reproduction by depressing fertility [13]. Furthermore, poor health and condition, and unbalanced energy budgets may compromise an individual's ability to evade predation. Effects in both top-down and bottom-up categories may exhibit density dependent and density independent characteristics. However, resource effects commonly exhibit density dependence at high abundance, whereas consumer effects are apt to be density independent at high abundance with possible density effects at low abundance [11].

Observations and physical evidence demonstrate the occurrence of predation on declining mesopredator populations in the GOA and BSIA [14]–[18], primarily by the transient ecotype of the killer whale [14], but also by salmon sharks (*Lamna ditropis*) and Pacific sleeper sharks (*Somniosus pacificus*) [12]. From a review of data collected through the 1990s the National Research Council concluded that recovery of the western Steller sea lion population was more likely limited by predation than by resource driven effects [11].

However, through indirect evidence interpreted in support of resource and other constraints on Steller sea lion productivity [19]–[22], [1], attention has recently shifted towards anthropogenic and natural bottom-up forcing, possibly expressed as reduced reproductive rates or natality. For the purposes of our study, we define natality as the number of female *and* male pups born divided by the number of females of reproductive age. Perhaps most influential in current discussions of sea lion population trajectories, Holmes and collaborators [21] used a time-varying Leslie population matrix to model vital rates for the central GOA region of the western Steller sea lion. Aerial survey photographs were used to estimate population age structure from animal length class distributions. Model demographic outputs were compared to observed abundances also obtained from aerial photographs. Fecundity schedules (adult females) and survival schedules (separately for juveniles and adults) from the central GOA region during the late 1970's used in the Leslie matrix were scaled by time periods to obtain best fits to observed population trajectories and juvenile fraction age structure metrics. Thus, the model yielded scaling factors by time periods for changes in natality, survivorship and age structure. From the best fitting inferential model the authors derived the following hypotheses: (1) natality steadily declined from 67% in the 1970's to 43% by 2006; (2) juvenile survival was depressed during the height of the decline in the mid 1980's; (3) juvenile survival recovered to pre-decline rates by 2006. Though only central GOA demographics were modeled, the authors proposed applicability of their hypotheses across the entire GOA and AI region.

However, all current hypotheses on forcing remain untested by direct measures, and factors driving past and present population trajectories are undetermined [1], [10], [22]–[24], largely due to immense logistical constraints on working with a large and effectively cryptic marine mammal, the Steller sea lion. Despite this absence of empirical testing, the vital rate changes proposed by Holmes et al. have been broadly embraced as the conceptual centerpiece of policy and management,, resulting in changes to research priorities and the recovery plan. In 2010, Maniscalco et al. [25] provided a direct assessment of Steller sea lion natality in the eastern GOA based on a longitudinal study of n = 151 individual females observed at the Chiswell Island rookery, and estimated natality at 69% (+/−2.5% S.E.). With their empirical evidence contradicting the hypothesized decline in natality at least for the eastern and likely central GOA region (Chiswell Island is located near the boundary between the eastern and central GOA regions), the authors concluded that ‘alternative hypotheses must be more seriously considered’ [25]. It is possible that the primary components of the past population changes may never be understood, but advances in tracking technology now provide an opportunity to directly measure aspects of sea lion biology that were previously ‘empirically intractable’ [17], specifically causes and rates of mortality.

To directly measure mortality and predation in the western Steller sea lion, we deployed specialized telemetry transmitters [26] in n = 36 juvenile sea lions from 2005 through 2011 in the Kenai Fjords and Prince William Sound region of the GOA. The abdominally implanted [27] archival Life History Transmitters (LHX tags) record data throughout the host's life. LHX tags primarily rely on sensor data from temperature, light, and dielectric properties of surrounding medium to determine host state [26]. After death, positively buoyant tags liberated from decomposing or dismembered carcasses, or passed through the digestive tract of predators will come to float on the ocean or rest ashore, and will then transmit stored data to orbiting satellites [26]–[28]. Transmitted data on light levels, surrounding medium, temperature profiles recorded across mortality events and time to transmission allow distinction of predation (rapid temperature drop, immediate sensing of air and light, immediate transmissions) from non-traumatic deaths (gradual temperature decline while surrounded by tissue, delayed sensing of light and air and onset of transmissions) [28]. To increase data recovery likelihood and estimate event detection probability, 34 of 36 animals received two implants.

We place our measures of post-weaning mortality and predation into the context of forcing by means of a qualitative conceptual framework. The framework integrates age structured, density dependent effects with density independent effects on survival, reproduction and population trajectories. From the combination of previously unavailable empirical data and qualitative conceptual framework we propose an alternative hypothesis to the postulated depressed natality for present day forcing of the Steller sea lion population in the eastern GOA.

## Materials and Methods

### Ethics Statement

This study was carried out in strict compliance with all applicable Animal Care and Use Guidelines under the U.S. Animal Welfare Act and was approved as required under the U.S. Marine Mammal Protection Act and the U.S. Endangered Species Act by the U.S. National Marine Fisheries Service (Permit numbers 1034–1685, 1034–1887, 881–1890, 881–1668, 14335, 14336) and by the Institutional Animal Care and Use Committees of the Alaska Sea Life Center (02-015, 03-007, 05-002, 06-001, 08-005, R10-09-04), and Texas A&M University (2003-181, 2005-170, 2006-37). All surgeries were performed under aseptic conditions and under full inhalant gas anesthesia, and all efforts were made to minimize pain and suffering.

### Animals, procedures and controls

Thirty six juvenile Steller sea lions (*Eumetopias jubatus*) were captured in Prince William Sound (PWS), Alaska from 2005 to 2011. Capture, transport to, temporary holding and husbandry at a quarantined facility at the Alaska SeaLife Center (Seward, Alaska) were performed as previously described [29], [30]. Intraperitoneal implantation of LHX tags (technical details in [26]) occurred under gas anesthesia using standard aseptic surgical procedures as previously described [27]. The first two animals received single LHX tag implants (2005), all subsequent animals received dual transmitter implants to facilitate estimation of data recovery probability. Post-operative clinical, physiological and behavioral monitoring lasted 1–6 weeks [27], [30]–[32]. All animals were released into Resurrection Bay in the Kenai Fjords area (2005 n = 2, 2006 n = 4, 2007 n = 5, 2008 n = 10, 2009 n = 6, 2010 n = 5, 2011 n = 4). All animals but one were hot-iron branded prior to release as per Mellish et al. [30]. All animals (male n = 28, female n = 8) were greater than 12 months of age and weaned at the time of capture. At the time of release, animals ranged from 13 to 22 months with the exception of one individual (25 months). Extensive post-surgical health assessments showed a typical mild to moderate immune and stress response to the procedure [30], [31]. All clinical health parameters monitored returned to baseline values within six weeks of surgery [30], [31], from which we derived a 45 day post-implant ‘confirmation of survival’ criterion for inclusion of individual animals in this study.

To confirm short-term survival, and to compare foraging and ranging behavior of LHX tag recipients to 23 non-implanted, temporarily captive control animals, 35 of the 36 LHX sea lions were monitored after release via externally attached, satellite-linked data transmitters [30], [33]. Minimum confirmed post-surgery survival was derived from external transmitter data, opportunistic re-sights of individual brands or LHX tag data over a range 47 to 2,072 days (mean 534+/−87.5 s.e.m., n = 36). All 36 study animals had survival confirmed beyond the 45 day criterion and are included in the results reported here. As previously reported, post release diving and ranging behavior did not differ between LHX tag recipients and non-implanted control animals [30], or between controls and free-ranging juveniles [33].

To evaluate potential long-term impacts of tags and surgeries on survival, we compared LHX-based survival rate estimates (see below) to rates derived from hot-iron brands applied to n = 255 juvenile sea lions in PWS by the National Marine Fisheries Service from 2001–2005, with re-sight surveys conducted from 2002–2008 (these data were evaluated using a Cormack-Jolly-Seber open population model in the program MARK [34], and were provided as sex specific annual survival rates and uncertainties by L. Fritz, pers. comm.). Sex-specific cumulative survival rates were obtained as products of sequential annual rates and weighted to reflect a similar sex ratio as the LHX study. Uncertainties were similarly obtained as weighted products of age and sex specific annual confidence limits.

### Estimation of mortality detection probability and survival rates

To estimate survival rates from LHX tag data, detected mortality events need to be corrected for events not detected due to failure of devices to successfully uplink to the ARGOS system aboard NOAA satellites. Uplink failures are the combination of technical tag failures and transmissions from a functional tag not reaching any satellite due to tag exposure constraints [26], [28]. Tag uplink failure probability was estimated from the ratio of single to dual LHX tag data returns from dual tag deployments (n = 34 live animal, n = 9 carcass test). The tag uplink failure probability *P*_fail_ can be calculated as *P*_fail_ = *C*_single_/(*C*_single_ + 2*C*_dual_) where *C*_single_ is the count of single returns, and *C*_dual_ is the count of dual returns. A correction factor *F* can then be derived as *F* = 1/(1-*P*_fail_^2^) and the corrected number of mortality events *E*_corr_ calculated as *E*_corr_ = *F* (*C*_single_ + *C*_dual_). From *P*_fail_ the event detection probability *P*_detect_ can in turn be derived as *P*_detect_ = 1-*P*_fail_^2^ = 1/*F* [28]. Ranges containing 95% of likely variance for the estimate of *P*_fail_ were derived from the Cumulative Distribution Function of a Monte Carlo simulation (>2,500 iterations) of randomly assigned individual tag failures for 0<*P*_fail-simulated_<1 yielding *P*_fail_ not exceeding the observed value without increasing *E*_corr_ integer counts. The 95% confidence range for *P*_fail_ in turn yields confidence intervals for *F* and *E*_corr_.

We calculated daily mortality rates from age-class specific subsets of cumulative exposure days (*d*_exp_) and corrected mortality counts as *DMR* = *E*_corr_/*d*_exp_. We then calculated daily survival rates *DSR* = 1 − *DMR*, and rates for periods longer than one day were obtained by raising *DSR* to the power of period duration in days (i.e. annual survival rates are *DSR* ^365.25^) [35], [36]. 95% confidence limits can be calculated from variance and standard error as per Johnson [36]. However, the Johnson method does not include the effects of *P*_fail_ on the estimation of *E*_corr_. To include the effects of uplink failures on estimation of survival rate confidence limits, we used the upper confidence limit for *E*_corr_ (and correspondingly reduced *d*_exp_) to re-compute the lower survival rate confidence limit as per Johnson (upper survival rate limits remain unchanged). Though a total of 29,581 exposure days were logged from 36 animals (ages of 13–90 months), complete year classes for >60 months were covered by only 5 animals (1,825 days for each year class) and inclusion of ages >60 months would substantially increase uncertainties. Therefore, only data from 24,072 cumulative exposure days over the ages of 13–60 months are used here (13–24 months: 35 animals×5,757 *d*_exp_; 25–36 months: 25 animals×7,763 *d*_exp_; 37–48 months: 18 animals×5,979 *d*_exp_; 49–60 months: 15 animals×4,573 *d*_exp_).

### Determination of causes of mortality

Causes of mortalities were inferred from temperature data recorded across mortality events, concurrent changes in surrounding medium (organic tissue, saltwater or air), time to sensing of light and onset of transmissions, and ancillary data as previously reported [28]. Gradual cooling and delayed extrusion are indicative of non-traumatic deaths (i.e. disease or starvation), or of entanglement, drowning or shooting. An *algor mortis* (body cooling) computational model parameterized for sea lions and validated through carcass testing allows the distinction of cooling masses if well outside of model uncertainties [28]. Precipitous drops to ambient temperature with immediate sensing of light and onset of transmissions are indicative of acute death by massive trauma associated with dismemberment by predators leading to the immediate release of tags [28]. Ship strikes, entanglement, drowning and shooting have been reported for the BSAI region [12]. Ship strikes on marine mammals are usually described as massive blunt force trauma but like drowning and shooting are unlikely to result in an immediate extrusion of both tags [28]. Thus, all acute and non-traumatic events *other than* predation should lead to a gradual transition to ambient temperatures as the entire carcass cools, with substantially delayed tag extrusion and onset of transmissions, unless the tags are liberated by immediate dissection.

To provide uncertainties for the estimated proportion of mortalities by predation *PP*, we conducted a Monte Carlo simulation of *n* mortalities (where *n* is the number of events detected for which cause of mortality could be determined) for simulated values of 0<*PP*<1 set in increments of 0.1 (10,000 iterations). The lower confidence limit was then calculated as 95% of the Cumulative Distribution Function of the actual number of predation events out of the number of detected mortalities.

### A simplified Leslie matrix to estimate age structured, cross sectional predation and minimum natality

To estimate cross-sectional, age structured consumption of sea lions by predators, we derived an updated contemporary survival schedule for a birth-pulse Leslie population matrix, separately for each sex (Table S1). The matrix uses a schedule of annual survival rates for ages from 1 month to 31 years, but excludes a fecundity schedule, since no accurate recent estimates exist for age specific adult female fecundity (see introduction). In a standard Leslie matrix, fecundity is used to estimate pup production and overall natality. Sequential matrix years are then seeded with pup production from preceding years to generate outputs for time-varying simulations of population trajectories [37], [21]. By excluding fecundity, the simplified matrix cannot be used to model time-variant population trajectories. However, even without fecundity the simplified matrix can be used to estimate *minimum natality* for conditions of stable or increasing population trajectories. The *minimum natality* yields an *equilibrium survivorship schedule* with a theoretical Eigenvalue of 1 for a corresponding time-variant matrix. Assuming that primiparity occurs at the average age of 5.3–5.6 years (after first ovulation at 4.3–4.6 yrs; [38], [25]), and that females reproduce through the age of 21 years but not beyond, *minimum natality* is estimated as the pup seed count divided by the number of females between the ages of 5 and 21 (inclusive). This assumed reproductive age span for adult females is consistent with the fecundity schedule used by Holmes et al. [21]. This simplistic measure does not require assumptions about age specific fecundity (i.e. any decline in natality with parity for old females), and the estimate is therefore independent from the age structure of the adult population, but is only applicable to stable or increasing populations.

We modified the best fit survival schedule from Appendix C of Holmes et al. [21] as adjusted by the authors using their best fit scaling factors for the 1998–2006 period. This schedule is denoted HFYS-06 in Table S1. The original, un-scaled survival schedule for pre-decline conditions used by Holmes et al. is also listed as HFYS-Pre. We modified the HFYS-06 schedule with survival rates for ages 13–60 months replaced by LHX-based estimates. We also replaced survival rates for ages 1–12 months with values averaged from Pendleton et al. [39] and Maniscalco et al. [40], [41]. This value used for young-of-the-year matches brand-resight based estimates by the National Marine Fisheries Service (L. Fritz, pers. comm.). Thus, survival rates in HFYS-06 for ages 1–60 months (the youngest 5 years) were replaced with more recent and location-specific estimates. This replacement however resulted in an improbably high *minimum natality* estimate of 0.92 compared to 0.6 for the unmodified HFYS-06 matrix (Table S1). To correct for this shift, we altered the scaling factor for adult survival from 1.07 (as used by Holmes et al. [21]) to 1.13 to produce a minimum natality estimate of 0.69, the value reported by Maniscalco et al. for the eastern Gulf of Alaska [25]. This resulted in the LHX-eGOA schedule listed in Table S1. The LHX-eGOA schedule uses identical values for males and females for ages 1–60 months, and assumes a 1∶1 sex ratio at birth. For males >60 months a survival schedule was generated as a progressively decreasing proportion of female rates to match sex-specific age frequency distributions to values reported for n = 235 males and n = 282 females collected from 1976–1989 by Calkins and Pitcher [42]. The sex specific schedules resulted in 95% of females in the population comprised within ages 1–19 years, and 95% of males within ages 1–14 years.

We added age-class specific estimates for the proportion of mortality by predation *PP* (see above), and from that in turn derived two mortality schedules separately for each sex, one for predation and one for all other sources of mortality. We used our LHX-based *PP* estimate for juveniles (ages 13–60 months). For young-of-the-year (ages 1–12 months) we used 30% of the *PP* value for juveniles to obtain predation rates comparable to values reported for the eastern GOA in literature [40]. For age classes >60 months we reduced the juvenile *PP* by 50% *p.a.* to account for a hypothesized reduction in vulnerability to predation with increasing age, size and experience (i.e. [17], [43], and see discussion). This resulted in adult predation accounting for only 4% of all predation events in females and 5% in males. As in a standard Leslie matrix, population vectors with absolute counts of animals in each age class can be generated from a birth-pulse (seed count of pups) and the survival schedule. Similarly, predation and non-predation vectors can be generated from the mortality schedules listed in Table S1.

### A conceptual, qualitative population model to combine density-dependent with density-independent effects

To evaluate the potential impact of the observed levels of post-weaning mortality by predation, we constructed a qualitative conceptual population model to integrate age structured, density dependent consumption of sea lions by predators with density independent mortality by other causes. We used the contemporary LHX-eGOA schedules (Table S1) to calculate vectors for population numbers, consumption by predators and non-predation mortalities for specific abundance levels (vector sums) from 0% to 100%. Population vector sums were adjusted through selection of appropriate pup seed counts (birth pulses) with 100% abundance set to the peak historic level of approximately 180,000 animals, and the recent population estimates of approximately 36,000 animals used for the 20% contemporary abundance (western U.S. stock [1], [44]). Non-predation mortality was assumed not to vary with density and the ***m_npi_*** schedule and corresponding mortality vectors therefore remained identical for all abundance levels. Numerical consumption by predators was adjusted as a function of sea lion abundance according to three different, age-structured response types (*Flat, Linear, Sigmoid*), yielding adjusted predation vectors. From the combined predation and non-predation vectors, an updated survivorship schedule was computed. Since this density-dependent model is not a time-variant Leslie matrix and has no fecundity schedule, a population trajectory cannot be calculated. However, from the sum of the female population vector for the ages of reproductive maturity (5–21 years, see above) multiplied by an assumed birth rate pup production can be estimated. We calculated the *potential trajectory* for a given abundance as the difference of the birth pulse seed count minus the actual pup production for a set birth rate.

A number of additional metrics were computed for comparative purposes: the total number of animals consumed for a given abundance (the numerical response) was calculated as the sum of the predation vectors for both sexes. An estimation of total sea lion mass consumed by predators was obtained by multiplying the predation vectors with an age-structured mass schedule separately for each sex. We used the mass schedules from Table 3 of Winship et al. [45], and used a mean mass of pregnant and non-pregnant females of reproductive age weighted by the proportion of females pregnant (the set birth rate). The juvenile fraction *J/T* was calculated as the population vector sums for ages 2–5 years, divided by the population vector sums for ages 2–31 years, for both sexes. Female recruitment was calculated as the percentage of the female seed count surviving to the end of year 4.

## Results and Discussion

### Detected mortalities and survival rates

Data from twelve detected mortality events were received from November 2005 through November 2011 during 24,072 cumulative exposure days. Seven events occurred within ages 13–24 months, four events within 25–36 months, and one event at age 49 months. From the ratio of dual LHX tag data returns (n = 9 from 12 detected mortalities plus n = 8 from 9 carcass simulations) to single returns (n = 3 live plus n = 1 carcass), we estimated tag uplink failure at *P*_fail_<0.105 and mortality event detection probability at *P*_detect_>0.989 (95% c.i. 0.0.92–1.0). Within these returns, the joint probability distribution of live animal and carcass returns gave an odds ratio of 2.7 (odds ratio test [46]), suggesting no differences in detection probabilities between live animal and carcass deployments (Fisher Exact Probability P_(2,1)_ = 0.6 [47]). From the correction factor *F* = 1.0112 we derived the corrected mortality count of *E*_corr_ = 12.13 (95% c.i. 12–13). However, since animals cannot die by fractional numbers, we used the integer portion of *E*_corr_ = 12 for subsequent calculations.

We estimated the cumulative survival for ages 13–36 months at 0.531 (95% c.i. 0.40–0.63, Table 1). This combined rate for both sexes is slightly lower than the control estimate of 0.534 based on brand re-sights but with overlapping confidence limits of (95% c.i. 0.42–0.64). Our annual estimates are on either side of controls (in parentheses) for subsequent year classes: 13–24 months: 0.641 (0.690), 25–36 months: 0.829 (0.775), 37–48 months: 1.0 (0.884); 49–60 months: 0.923 (0.875). Our estimate for the cumulative survival for ages 13–60 months is 0.491 (Table 1). Since LHX tags deliver event data with a resolution <1d, survival rate estimation only requires inferences on dates of undetected events, less than 3% of events or 1.5% of animals in our study. Brand re-sight based survival estimates require inferences on dates for all apparent mortalities, more than 50% in the control study by the National Marine Fisheries Service. For annual rates or re-sight efforts unknown dates are inferred to +/−182.5d. This difference explains the comparable uncertainties for these two distinct methods with sample sizes that differ by almost one order of magnitude. Furthermore, LHX tags provide information not only on dates, but on locations and causes of mortality of individual animals. Within the limits of the uncertainties for both techniques, the survival rate comparison does not suggest any impact of LHX tags, surgeries or temporary captivity on survival for ages 13–60 months.

**Table 1:** Cumulative survival for juvenile Steller sea lions for ages 13 through 60 months estimated by different methods.

Model and Period	13–36 months	13–48 months	13–60 months
LHX-eGOA 2005–2010	0.531 (0.40–0.63)	0.531 (0.43–0.60)	0.491 (0.40–0.54)
HFYS-06 1997–2006	0.72 (0.70–0.77)	0.67 (0.65–0.72)	0.61 (0.59–0.66)
HFYS 1983–87	0.42 (0.40–0.47)	0.39 (0.37–0.44)	0.36 (0.33–0.40)
HFYS-Pre 1976–82	0.75	0.70	0.64

Model LHX-eGOA is based on LHX transmitter data returns. Models HFYS are from a published inferential model [21] for three distinct time periods. Numbers in parentheses are 95% confidence intervals where available.

Mortalities occurred in two of eight monitored females and ten of 28 monitored males. The odds ratio was 1.43, suggesting no differences in mortality probabilities between sexes within the data set (Fisher Exact Probability P_(2,1)_ = 1.0). Mortalities occurred 1 each in August, September, October, November and March; 2 each in January and May; and 3 in February.

**Figure 1:**
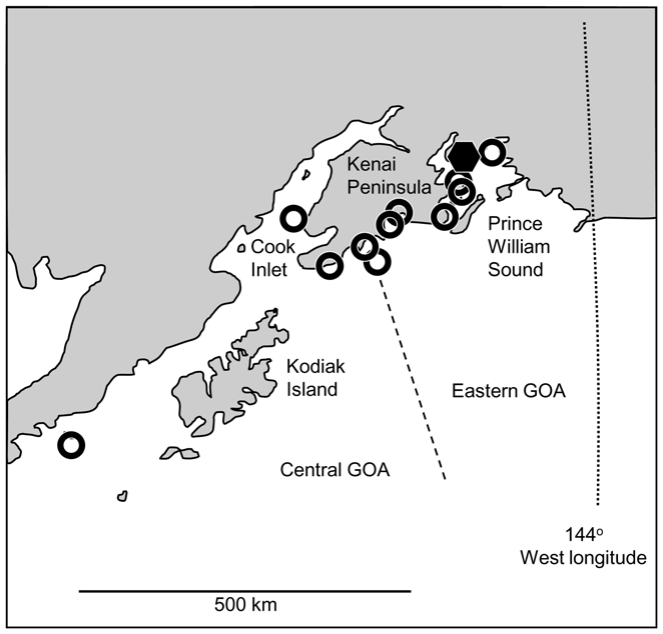
Locations of juvenile Steller sea lion mortalities detected in the Gulf of Alaska. Eleven predation events indicated by open circles occurred in the eastern and central Gulf of Alaska (GOA). One mortality of undetermined cause indicated by the solid hexagon occurred in Prince William Sound. The endangered Western Distinct Population Segment is located to the west of 144° longitude.

Event location accuracy from four events with pre-mortality locations from external tracking devices was estimated at 10.4 km (+/−3.1 s.e.m.) [28]. Eleven of the twelve detected mortalities occurred within the previously described geographic range covered by implanted and non-implanted juvenile Steller sea lions following release from temporary captivity [30], [33] (Fig. 1). One event occurred outside of this area, to the west of Kodiak Island, though this is still within known ranges of juvenile sea lions [48]. No mortalities occurred near rookeries, and four of the twelve events occurred near juvenile-dominated haulouts.

### Causes of mortality

One of twelve detected events (in age class 25–36 months) provided no data other than confirmation of mortality and event date, due to a technical failure in the single LHX tag of the implanted pair that successfully uplinked. Eleven events provided data including death time stamps, time from death (determined as per [26], [28]) to onset of transmissions, and complete 48 hour temperature profiles across the mortality events. Ten events exhibited precipitous temperature drops with immediate tag extrusion and onset of transmissions, indicative of predation. One event exhibited gradual cooling with *algor mortis* model outputs corresponding to 14% of predicted mass, suggesting partial dismemberment, most likely due to predation [28]. Thus, all eleven detected events that yielded data were classified as predation events. The combined probability distribution of single to dual tag returns for these eleven predation events and the nine carcass tests that constitute simulated non-traumatic deaths (2 in 9 for predation and 1 in 8 for non-traumatic deaths) gave an odds ratio of 0.56 suggesting no differences in detection probabilities between predation versus non-traumatic events (Fisher Exact Probability P_(2,1)_ = 1.0). The finding of a minimum of eleven predation events in twelve detected mortalities yields an estimated proportion of juvenile sea lion (ages of 13–60 months) mortalities in the PWS-KF region due to predation of *PP*> = 0.917 (95% c.i. 0.78–1.0). However, we used the center of the 95% confidence interval of *PP* = 0.89 for all subsequent model calculations. Given that most observations of Steller sea lions typically occur during the breeding season (June–September) with focus on rookeries [16], [40], [49]), the timing and location of our detected mortality events suggest that previously reported predation rates may be greatly underestimated.

### Density-dependent predation models

Predator-prey theory defines three primary types of density dependent prey consumption rates for a given predator density, the functional response [50]. Corresponding numerical responses characterize absolute prey consumption numbers. The Lotka-Volterra functional response (Type I) is primarily applicable to sedentary predators (i.e. web spiders). Type II (Hyperbolic) and Type III (Sigmoid) are both theoretically applicable to mobile marine apex predators and their prey, although their functional responses have not been empirically characterized. Other, less common functional response types exhibit distinct predator-prey dynamics at high densities [51] improbable for marine apex predators. Types II and III are comparable at high densities, but exhibit distinct predator-prey relationships at medium and low prey densities [50]–[52]. The Hyperbolic response (Type II) is applicable to specialist predators focusing on a single prey species and should result in accelerating prey declines at lower densities which may lead to extinction [52]. The Sigmoid functional response (Type III) is applicable to non-specialist predators that can shift to alternate prey at low prey densities. The Type III functional response results in diminishing prey removal rates at very low densities as predators increasingly switch to alternate prey, in turn creating a prey ‘refuge’ with increasing survival rates. By comparison, predator-prey systems comprised of pelagic mesopredator fishes and their prey have been studied, and dynamics likely differ from those of marine homeotherms and their apex predators. The dynamically linked populations with density feedbacks of specialized pelagic fishes and their prey are more commonly characterized through a combination of aggregative and numerical responses [51], [53], [54].

**Figure 2:**
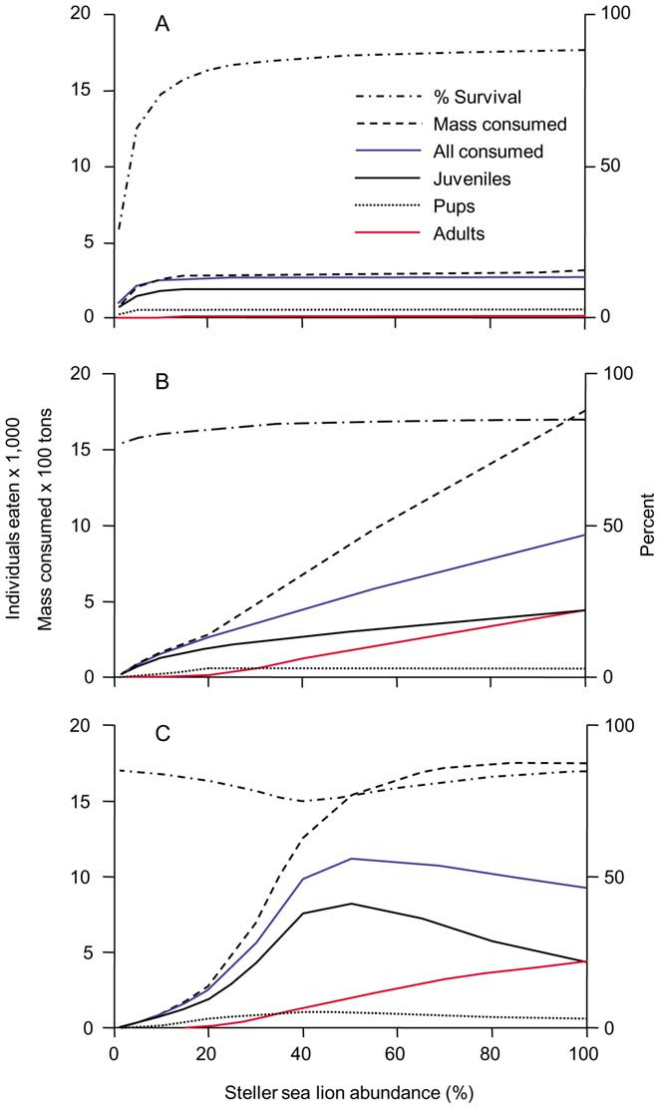
Survival and consumption by predators modeled as a function of Steller sea lion abundance. The percentage of all sea lions surviving to the end of a year, as well as the total mass and numbers of individuals consumed by predators are shown as a function of sea lion abundance, for three distinct numerical response types (see methods and discussion). (A) The *Flat* numerical response. (B) The *Linear* numerical response. (C) The *Sigmoid* numerical response. Numbers of individuals consumed are separately shown for pups (year 1), juveniles (years 2–4) and adults (years 5–31). For the western population, 100% abundance corresponds to 180,000 individuals.

We considered three distinct types of numerical responses between Steller sea lions and their predators. A *Flat* response was generated by not altering the overall and age-class specific prey consumption amounts for abundance levels above 20% (Fig. 2A). The *Flat* numerical response could occur for any type of functional response (Type I, II or III) under the assumption that resource needs of the predator population(s) are fully met at the 20% prey abundance level and saturated above that. A *Linear* response was generated by linearly increasing age-class specific prey consumption amounts from estimates at 20% abundance levels to estimates for the 100% abundance level (Fig. 2B). Consumption estimates for the 20% level were derived as described under methods from the LHX-eGOA schedule. Estimates for the 100% level were derived by setting age-class specific consumption amounts such that the resulting cumulative survival rates for ages 1–5 and 6–10 years matched the HFYS-Pre survivorship schedule (Table S1). The *Linear* numerical response corresponds to a Lotka-Volterra (Type I) functional response rarely seen in non-sedentary predators, but that could occur for highly opportunistic, non-aggregating pelagic predators that pursue a very large variety of prey species, such as sharks. A *Sigmoid* numerical response was generated as a modification of the *Linear* response, by increasing the consumption of juveniles and pups in relation to adults for abundances between 20% and 100% in order to maintain a flat combined consumption of mass for high abundances (Fig. 2C). The *Sigmoid* numerical response corresponds to a sigmoid functional response (Type III) that should be applicable to non-specialist predators such as transient killer whales and possibly sharks. The largely stable mass consumption from 100% down to 60% abundance in our model is concomitant with an increasing consumption of juveniles to compensate for a declining consumption of adults. This differential response by age classes (Fig. 2C) should be driven by the greater vulnerability to predation of younger age classes balanced against the reduced profitability in the form of lower individual mass and energy content (see [17], [43]). From an energy content analysis of Steller sea lions, Williams et al. [17] estimated caloric requirements of adult killer whales at 2–3 Steller sea lion pups per day versus one adult female sea lion every 2–3 days. No data exist on the energetic cost or risk of killing and consuming an adult Steller sea lion versus a pup or juvenile. However, the notion of age-structured predation pressure is conceptually supported through a risk-benefit model specifically developed for juvenile Steller sea lions, killer whales and Pacific sleeper sharks by Frid et al. [43]. This model explained seasonal differences in telemetered dive behavior of juvenile Steller sea lions in PWS through a combination of resource distribution, and higher predation pressure on juveniles than older sea lions. Empirical support for age structured predation pressure on pinnipeds in general is available for Hawaiian monk seals (*Monachus schauinslandi*). From an analysis of 315 shark-inflicted injuries recorded during an 11 year period Bertilsson-Friedman [55] concluded that sharks injured more pups and juveniles than subadults and adults. LHX tag data provides direct evidence of elevated predation risk for younger juveniles at current abundance levels. Since all detected events with sufficient data were classified as predation, the predation probability is the inverse of annual survival rates, or 35.9% for ages 13–24 months, 13.2 to 17.1% for ages 25–36 months depending on actual cause for the single unknown fate event, zero for 37–48 months and 7.7% for ages 49–60 months.

As the density of more profitable adults decreases, juveniles may become increasingly viable alternate prey in a form of intraspecific diet shift. While most marine mesopredators and their prey are distributed in the three-dimensional pelagic zone, the distribution of pinnipeds is constrained in space and time through reproductive activities tied to solid substrate ashore or on ice. This constraint is not uniform across sex and age. Young Steller sea lions are suckled by females at shore-based rookeries and haul-outs, and are typically weaned by the age of one year, with some documented cases of maternal care extended through the ages of 2 or 3 years [38], [56]. This results in juvenile age classes (ages 1–36 months) and adult females with increased spatio-temporal constraints in the form of predictable presence near specific shore locations. Within our model, the notion of density dependence of vulnerability and/or spatio-temporal accessibility of juveniles is supported by a comparison of consumption rate estimates between 20% and 100% abundance (Fig. 2C). This comparison is based on the LHX-eGOA (20%) and HFYS-Pre (100%) schedules under the central assumption in our model that non-predation mortality is density independent. At 100% abundance, pups comprise 7% of predation events, juveniles 46% and adults 47%, whereas at 20% abundance, pups comprise 23%, juveniles 72% and adults 5%. This changeover strongly suggests an age structured density dependence in predation rates.

### The likely numerical response

**Figure 3:**
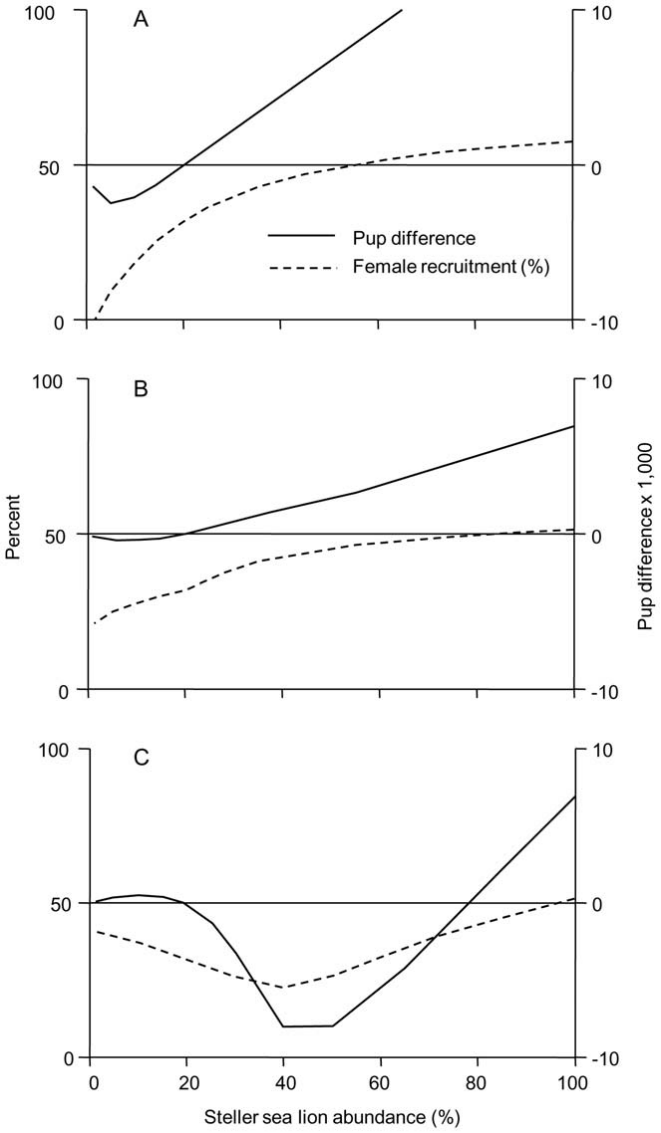
Potential Steller sea lion population trajectory and female recruitment modeled as a function of abundance. The potential population trajectory is calculated as the difference between the birth pulse pup seed count (the theoretical requirement for a stable population) and the actual number of births for a given natality and survivorship schedule - the Pup difference - see text. Female recruitment is the percentage of female pups surviving to the end of year 4. 100% abundance corresponds to 180,000 individuals. (A) The *Flat* numerical response. (B) The *Linear* numerical response. (C) The *Sigmoid* numerical response.

**Figure 4:**
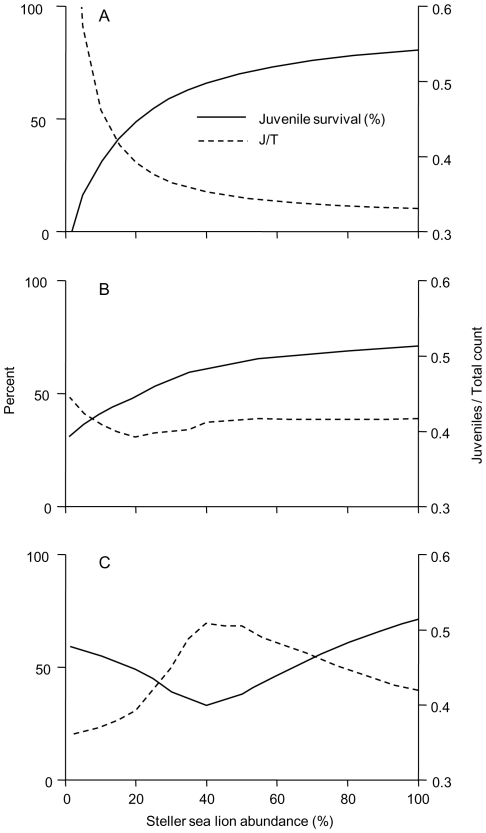
Juvenile survival and the juvenile fraction modeled as a function of Steller sea lion abundance. Juvenile survivorship is shown as the percentage of all juveniles ages 2–4 years that survive to the end of a year. The J/T metric is the count of all juveniles ages 2–4 years divided by the count of all ages 2–31 years (excluding only pups). (A) The *Flat* numerical response. (B) The *Linear* numerical response. (C) The *Sigmoid* numerical response.

The Sigmoid numerical response emerges as the most likely scenario for Steller sea lions and their predators for the following reasons: **(1)** Presently, the western population overall is stable at about 20% of peak abundance [1], [44]. The *Sigmoid* model is the only scenario to result in stable trajectories at 20% following a decline (as indicated by the negative slope of the pup difference curve at 20% abundance in Fig. 3). The *Flat* and *Linear* models both exhibit continuing declines. **(2)** Contemporary juvenile survival has only slightly recovered from lowest levels around 40–50% abundance, but is still below peak abundance levels (Table 1). This is the pattern seen in the *Sigmoid* model (Fig. 4C). Both *Flat* and *Linear* models show accelerating declines in juvenile survival with decreasing abundance (Fig. 4A,B). **(3)** The juvenile fraction metric (J/T in Fig. 4) is perhaps the most interesting comparative criterion. Holmes and York [57] provided a retrospective analysis of the juvenile fraction based on length measurements conducted on aerial survey photographs of rookeries and haulouts, for the central GOA. Though their actual ratios based on hauled out animals may differ from our comprehensive estimates for all animals, they reported an increase in the J/T ratio from early- to peak decline, followed by a decrease. This pattern is seen in the *Sigmoid* model, which exhibits a peak in the J/T ratio between 40 and 50% abundance, concurrent with the lowest juvenile survival rates and the greatest pup deficit. The *Flat* and *Linear* models conversely show a highly improbable continuing and progressively steeper increase in the J/T metric (*Flat*) or minimal changes down to 40% followed by a very slight drop to a minimum near 20% abundance (*Linear*). Thus, of the three response types considered, the age-structured Sigmoid type is the one most consistent with all available contemporary and retrospective demographic data.

At the contemporary 20% abundance level, model outputs suggest an annual consumption of 2,676 western Steller sea lions or 288 metric tons. This increases to near 11,300 sea lions eaten at 50% abundance or 1,759 tons consumed annually at peak abundance (Fig. 2C). A simple estimate suggests that these numbers are plausible: combining the current population estimate of 345 transient, mammal eating killer whales for the GOA/BSAI region [58] with the lower of two published theoretical sea lion consumption rates (exclusive of other prey) from caloric requirement estimates [17], [40] yields a potential consumption of over 100,000 juvenile Steller sea lions per year. Thus, only about 10% of transient killer whale diet would have to comprise Steller sea lions to yield the modeled effects. The steep increase from 1,938 juveniles consumed annually at 20% abundance to a peak of 8,240 juveniles eaten at 50% abundance shown in Figure 2C may appear as improbably high. However, this increase corresponds to a maximum *PP* of 0.92 at 40% abundance – well within the confidence limits of our contemporary estimate, and a minimum annual survival rate for the most vulnerable age class of 13–24 months of 49%. The latter is within the confidence limits of our contemporary estimate, and still above the estimated juvenile survival for the height of the decline as per York [37].

### Implications of a Type III numerical and functional response

Our framework suggests female recruitment as a key mechanism by which predation may drive the overall reproductive output and the potential trajectory function. From a positive potential trajectory at peak abundance down to the greatest pup deficit at 40–50% abundance, the percentage of adult females directly consumed by predators actually drops from 3.6% to 3.5%, but female recruitment concurrently drops from 51% to only 23% (Fig. 3C). Even without any changes in natality high predation on juveniles could effectively cut the reproductive potential of the population in half. Even if actual natality were to increase above the contemporary regional estimate of 0.69 [25] to 1.0, this would merely shift the equilibrium density from 20% to about 32% abundance. At higher abundance, the potential trajectory function would remain negative. Only significantly reduced consumption of juveniles at intermediate densities would lead to a positive potential trajectory at all abundances greater than 20%, and to full recovery. Rather than resulting in a plain predator pit from direct consumption of sea lions, the age-structured sigmoid response yields a *predation-driven productivity pit* mediated by female recruitment from which the population cannot escape even at a theoretical natality of 1.0 without reduced predation pressure.

Even though our density-dependent conceptual framework with age-structured predation rates provides the most parsimonious explanation for all observed vital rate dynamics, it is important to consider that actual dynamics could be heavily influenced by other factors affecting non-specialist predators. In particular at low abundance levels, numerical consumption of sea lions may be subject to the availability of alternate prey. Considering the broad scale declines of many upper trophic marine vertebrates in the Gulf of Alaska, Aleutian Island and Bering Sea region (see introduction) the applicability of a Type III response at and above 20% abundance may not necessarily result in reduced predation at lower abundance that typically provides a refuge from predation in a predator pit scenario [52], [53]. In addition, over broad periods of time such as the western Steller sea lion decline both non-predation causes of mortality and natality may vary. Within our framework predation at the levels estimated for 100% abundance alone would not initiate a decline (Fig. 3C). However, at the estimated pre-decline natality of 0.63 [21] a decrease in overall, cross-sectional survival from 85.1% to 83.1% could initiate a decline. Such a reduction in overall survival and thus the early decline could have been initiated by a comparably small reduction in carrying capacity [9], [59].

### Conclusions

Our data and model do not support the hypotheses derived from extant age-structured demographic models for the western Steller sea lion of recently recovered juvenile survival and depressed reproductive rates, for our study area. Instead, our data demonstrate continued low juvenile survival in the Prince William Sound/Kenai Fjords region of the Gulf of Alaska, and indirectly confirm recently published empirical studies in support of high reproductive rates. Our results on contemporary predation rates combined with a density dependent conceptual framework suggest predation on juvenile sea lions as the largest impediment to recovery of the species in the eastern Gulf of Alaska region. Our data and model do not however allow the determination of historic causes of the decline, nor of primary factors driving population trajectories outside of the study region. Nevertheless, our conceptual framework generally suggests the distinct possibility of predation as a major component of Steller sea lion population dynamics in particular at intermediate and low abundance levels. The framework also highlights the necessity for demographic models based on age-structured census data to incorporate the differential impact of predation on multiple vital rates, in order to gain credibility. As highlighted by Boyd in 2010 [59], the applicability of extant population models fitted to census data are limited by unknown and non-stationary error structures within datasets, including population structure data. The empirical validation of the functional and numerical response applicable to Steller sea lions and their predator and the impact of the availability of alternate prey (to the sea lions' predators) thus emerge as key research objectives in particular for regions of continuing decline.

## Supporting Information

Table S1 **Contemporary Steller sea lion vital rate schedules for the eastern Gulf of Alaska (LHX-eGoA).** The age classes listed (*i*) comprise months 1 through 12 for the first year, months 13 through 24 for the second year, and so forth. The survivorship schedules *s_i_* list the proportion of animals that were alive at the beginning of each year, that survive to the end of year *i*. *PP_i_* is the proportion of mortality (1-*s*) attributed to predation, for each year *i*. Mortality schedules *m_i_* list the proportion of animals consumed by predators (*p*) and those that died from other causes (*np*) by the end of each year *i*. The minimum birth rate *N_min_* (for definition see methods) for an equilibrium survivorship schedule is listed. Also listed are two survivorship schedules from Holmes et al. [21] for pre-decline conditions (HFYS-Pre for 1976–1982), as well for the 1998–2006 period (HFYS-06).
(DOC)
