# Supplementary material for: Predation on an Upper Trophic Marine Predator, the Steller Sea Lion: Evaluating High Juvenile Mortality in a Density Dependent Conceptual Framework
Source: PLoS One. 2012 Jan 17;7(1):e30173. doi: 10.1371/journal.pone.0030173 (PMC3260237; doi:10.1371/journal.pone.0030173)
Supplement: Table S1 — Contemporary Steller sea lion vital rate schedules for the eastern Gulf of Alaska (LHX-eGoA). The age classes listed (i) comprise months 1 through 12 for the first year, months 13 through 24 for the second year, and so forth. The survivorship schedules si list the proportion of animals that were alive at the beginning of each year, that survive to the end of year i. PPi is the proportion of mortality (1-s) attributed to predation, for each year i. Mortality schedules mi list the proportion of animals consumed by predators (p) and those that died from other causes (np) by the end of each year i. The minimum birth rate Nmin (for definition see methods) for an equilibrium survivorship schedule is listed. Also listed are two survivorship schedules from Holmes et al. [21] for pre-decline conditions (HFYS-Pre for 1976–1982), as well for the 1998–2006 period (HFYS-06). (DOC) [file pone.0030173.s001.doc]

Table S1. Contemporary Steller sea lion vital rate schedules for the eastern Gulf of Alaska (LHX-eGoA). The age classes listed (i) comprise months 1 through 12 for the first year, months 13 through 24 for the second year, and so forth. The survivorship schedules si list the proportion of animals that were alive at the beginning of each year, that survive to the end of year i. PPi is the proportion of mortality (1-s) attributed to predation, for each year i. Mortality schedules mi list the proportion of animals consumed by predators (p) and those that died from other causes (np) by the end of each year i. The minimum birth rate Nmin (for definition see methods) for an equilibrium survivorship schedule is listed. Also listed are two survivorship schedules from Holmes et al. [21] for pre-decline conditions (HFYS-Pre for 1976 – 1982), as well for the 1998 – 2006 period (HFYS-06).
Age class	LHX-
eGoA	HFYS-Pre	HFYS-
06	
i	si	si	PPi	mpi	mpi	mnpi	mnpi	si	si	
	female	male		female	male	female	male	female	female	
1	0.650	0.650	0.263	0.093	0.093	0.257	0.257	0.806	0.788	
2	0.641	0.641	0.875	0.320	0.320	0.039	0.039	0.847	0.829	
3	0.829	0.829	0.875	0.152	0.152	0.019	0.019	0.889	0.869	
4	1.000	1.000	0.875	0.000	0.000	0.000	0.000	0.930	0.932	
5	0.923	0.923	0.875	0.069	0.069	0.008	0.008	0.909	0.911	
6	0.947	0.929	0.438	0.023	0.032	0.029	0.040	0.895	0.897	
7	0.936	0.917	0.219	0.014	0.018	0.050	0.065	0.884	0.886	
8	0.926	0.907	0.109	0.008	0.010	0.066	0.082	0.875	0.877	
9	0.917	0.890	0.055	0.005	0.006	0.078	0.104	0.867	0.869	
10	0.910	0.873	0.027	0.003	0.004	0.088	0.123	0.859	0.861	
11	0.903	0.857	0.014	0.001	0.002	0.096	0.141	0.853	0.855	
12	0.896	0.843	0.007	0.001	0.001	0.103	0.156	0.847	0.849	
13	0.890	0.828	0.003	0.000	0.001	0.109	0.171	0.841	0.843	
14	0.885	0.814	0.002	0.000	0.000	0.115	0.186	0.836	0.838	
15	0.880	0.801	0.001	0.000	0.000	0.120	0.199	0.831	0.833	
16	0.875	0.787	0.000	0.000	0.000	0.125	0.212	0.827	0.829	
17	0.870	0.766	0.000	0.000	0.000	0.130	0.234	0.822	0.824	
18	0.866	0.745	0.000	0.000	0.000	0.134	0.255	0.818	0.820	
19	0.862	0.724	0.000	0.000	0.000	0.138	0.276	0.814	0.816	
20	0.858	0.703	0.000	0.000	0.000	0.142	0.297	0.811	0.812	
21	0.854	0.683	0.000	0.000	0.000	0.146	0.317	0.807	0.809	
22	0.850	0.663	0.000	0.000	0.000	0.150	0.337	0.803	0.805	
23	0.847	0.644	0.000	0.000	0.000	0.153	0.356	0.800	0.802	
24	0.843	0.624	0.000	0.000	0.000	0.157	0.376	0.797	0.799	
25	0.840	0.605	0.000	0.000	0.000	0.160	0.395	0.794	0.796	
26	0.837	0.586	0.000	0.000	0.000	0.163	0.414	0.791	0.793	
27	0.834	0.567	0.000	0.000	0.000	0.166	0.433	0.788	0.790	
28	0.831	0.548	0.000	0.000	0.000	0.169	0.452	0.785	0.787	
29	0.828	0.530	0.000	0.000	0.000	0.172	0.470	0.782	0.784	
30	0.825	0.512	0.000	0.000	0.000	0.175	0.488	0.780	0.781	
31	0.822	0.493	0.000	0.000	0.000	0.178	0.507	0.777	0.779	
32	0.000	0.000	0.000	0.000	0.000	1.000	1.000	0.000	0.000	
Nmin	0.690							0.574	0.604	
